# Correlation between TNF-*α* -308 and +489 Gene Polymorphism and Acute Exacerbation of Chronic Obstructive Pulmonary Diseases

**DOI:** 10.1155/2021/6661281

**Published:** 2021-03-01

**Authors:** Suyun Yu, Min Xue, Zhijun Yan, Bin Song, Haiping Hong, Xiwen Gao

**Affiliations:** ^1^Department of Respiratory, Minhang Hospital, Fudan University, Shanghai 201199, China; ^2^Department of Radiology, Minhang Hospital, Fudan University, Shanghai 201199, China; ^3^Shanghai Songjiang District Fangta Hospital of Traditional Chinese Medicine, Shanghai 201699, China

## Abstract

Acute exacerbation of chronic obstructive pulmonary disease (AECOPD) is becoming a common respiratory disease, leading to increased morbidity and mortality worldwide. Tumor necrosis factor-alpha (TNF-*α*) is a powerful proinflammatory cytokine involved in the pathogenesis of AECOPD. Therefore, we proposed a close correlation between the TNF-*α* polymorphism [-308G/A (rs1800629), +489G/A (rs1800610)] and the disease progress of patients with AECOPD. Comparison of the TNF-*α* genotypes between the 198 AECOPD diagnosed patients groups and 195 healthy peoples suggested their significant differences of the three genotypes (AA, GA, GG) distribution for TNF-*α* -308 (*P* < 0.05), but no differences of that for TNF-*α* +489. We found that patients with TNF-*α* -308 GA/AA genotypes showed smaller adjacent arterial diameter, thicker bronchial wall, higher bronchial artery ratio, higher bronchial wall grading, and higher frequency of acute exacerbations than those with TNF-*α* -308 GG genotype. Patients with TNF-*α* +489 GA/AA genotypes showed the same AECOPD properties as patients with TNF-*α* -308 except for the high frequency of acute exacerbations. Further experiment showed that the TNF-*α* -308 and+489 gene polymorphisms could affect the expression level of TNF-*α* in macrophages, suggesting the involvement of the macrophage population in disease regulation of AECOPD patients with TNF-*α* -308G/A and+489G/A genotype heterogeneity. In conclusion, the TNF-*α* -308 G/A genotype was related to AECOPD susceptibility and progress, while the TNF-*α* +489G/A genotype was related to AECOPD progress, but not AECOPD susceptibility.

## 1. Introduction

Chronic obstructive pulmonary disease (COPD) refers to a group of chronic inflammatory diseases that cause airflow blockage and breathing-related problems [1, 2]. Although causing high morbidity and mortality, there is no specific treatment for COPD, and the pathogenesis remains unclear. COPD can develop into acute exacerbation of COPD (AECOPD). The patients with AECOPD suffer from rapid deterioration of the lung, accompanied by inflammation and fever. Frequent recurrence of AECOPD is an important factor in promoting the progression of COPD, leading to increased morbidity and mortality worldwide [3–5]. Smoking is previously shown to be significant associated with AECOPD; however, it is reported that only 10-15% of smokers could develop AECOPD [6, 7]. Besides, there also appears to be a familial clustering of AECOPD [8]. These studies suggest that genetic factors may play an important role in AECOPD development. Therefore, exploring the association of heterogeneity of AECOPD phenotypes and gene polymorphism is necessary for individualized prevention and treatment programs.

Macrophages are the most abundant cells in the tumor stroma and exhibit obvious plasticity, which enables them to perform multiple functions in the tumor microenvironment [9]. Tumor-associated macrophages usually refer to another type of M2, which exhibits anti-inflammatory and tumor-promoting effects [10]. In large series of nonresponding community-acquired pneumonia (CAP) patients, COPD is observed to be a protective factor for nonresponse to initial antibiotics [11]. This result is related to changes in the phenotype of inflammatory cells, especially the induction of classic M1 or alternative M2 activation of macrophages is involved, which leads to different inflammatory conditions [12]. Previous studies indicate that the microenvironment in the lung regulates the activation of macrophages, resulting in different phenotypes in AECOPD, CAP, and COPD + CAP patients [11].

Tumor necrosis factor- (TNF-) *α*, working as a proinflammatory cytokine, plays an important role in an inflammatory response. Many studies show that TNF-*α* is upregulated in the sputum, broncho alveolar lavage fluid, and bronchial biopsies of patients with COPD [13, 14], suggesting the key role of TNF-*α* in the COPD progress. As a well-studied proinflammatory cytokine, many biallelic polymorphisms of TNF-*α*, including -308G/A (rs1800629), -376G/A (rs1800750), -238G/A (rs361525), and+489 (rs1800610) gene polymorphisms, are discovered. Among these polymorphic sites, -308G/A, -376G/A, and -238G/A polymorphisms are located in the promoter region of the gene, whereas +489 polymorphism is located in the first intron of the gene [15]. Many studies have assessed the relationship between COPD risk and TNF-*α* polymorphisms. As the best-studied TNF-*α* polymorphism [16, 17], -308 G/A variant has been reported to be closely related with COPD development in a Taiwanese [18] cohort and a Japanese cohort [19], but not in Caucasian populations [20–22]. Besides, TNF-*α* +489 G/A polymorphism, which is involved in prostate cancer, systemic lupus erythematosus, and rheumatoid arthritis development [23], is also recently identified to be related to COPD development [24, 25]. However, few studies are performed on the Asian population. Therefore, we perform a correlation analysis between TNF-*α* polymorphisms (-308 G/A and+489 G/A) and AECOPD susceptibility of the Shanghai Han Population.

## 2. Materials and Methods

### 2.1. Specimen Acquisition

The AECOPD (159 males and 39 females) patients and healthy populations (157 males and 38 females) studied in this work were obtained from the Department of Respiratory Medicine at Minhang Hospital, Fudan University. A total of 198 patients and 195 healthy peoples of the Shanghai Han population were selected from January 2014 to June 2015. The Research Ethics Committees of Minhang Hospital approved the study, and all patients provided written informed consent.

Specifically, patients were diagnosed as AECOPD according to the criteria described in “Chronic obstructive pulmonary disease treatment guidelines (2013 Revision)” established by the Chinese Medical Association Respiratory Diseases Committee. To be specific, eliminate other disease possibilities, after inhaling bronchodilator, FEV_1_/FVC < 70%; patient respiratory symptoms deteriorated more than the daily variation range, and when there is a need to change the drug regimen, during the disease, patients often had a short-term cough, sputum, shortness of breath and/or exacerbated wheezing, increased sputum, purulent or mucopurulent sputum, fever, and inflammation to significantly exacerbate symptoms. Patients with the genetic predisposition of other diseases, including hypertension, diabetes, coronary heart disease, or rheumatism, were excluded from the sample cohort. As for the control cohort, 195 healthy Han individuals (157 males and 38 females) without disease history of other respiratory or systemic diseases, such as bronchiectasis, pulmonary fibrosis, sarcoidosis, asthma, lung cancer, diabetes, and heart disease, were selected. All of the individuals were confirmed as the normal lung and chest function through high-resolution computerized tomography (HRCT) examination.

Detailed pathological datum, including medical history, age, gender, smoking index, and specific symptoms (cough, sputum, or dyspnea) was collected from the patients with AECOPD or the control subjects. Besides, the following two types of COPE classification were considered: Pistolesi et al. [26] revealed that based on the clinical manifestations, spirometry results, chest imaging data, and pathologic features, COPD could be divided into bronchitis- and emphysema-types: the criteria outlined by Hurst et al. [27], and AECOPD phenotypes can be divided into nonfrequent- (0-1 time/year-) and frequent- (≥2 times/year-) types. Spirometry was performed for all patients. The lung functions of all subjects were evaluated using the COSMED quark-PFT4 spirometer.

All patients underwent chest HRCT examination at the end of a calm breath, using a 64-slice helical CT scan, from the apex to the bottom of the lung. Scanning parameters were as follows: voltage 140 kV, thickness 1.25 mm, current 112 mas, pitch 1.375 : 1, and lap speed 12.25 s. Workstation syngo. via was used to read the sections.

### 2.2. Data Analysis

Two radiologists evaluated the clinical data under double-blinded conditions. The average values of all indicators were taken into analysis. Based on the principles using 960 HU to define the normal lung and low attenuation area (LAA) published by Kitagichi et al. [28, 29], the 3 anatomical levels at the edge of the aortic arch, lower spine, and pulmonary vein were evaluated, and the area of LAA on either side was calculated as a percentage of the whole lung. Subsequent comparison and classification were performed. The rating criteria were as follows: LAA < 5%, 1 point; 5% ≤ LAA < 25%, 2 points; 50% ≤ LAA < 75%, 3 points; and LAA ≥ 75%, 4 points. The 6 viewpoint scores were added to calculate the emphysema score: 0 points, stage 0; 1 to 6 points, stage I; 7 to 12 points, stage II; 13 to 18 points, stage III; 19 to 24 points, and stage IV. Bronchial wall thickness was graded as follows: 0, bronchial wall thickness < 30% of the adjacent pulmonary artery diameter; grade 1, 30% of the adjacent pulmonary artery diameter ≤ bronchial wall thickness < 50% of the adjacent pulmonary artery diameter; and grade 2, bronchial wall thickness ≥ 50% of the adjacent pulmonary artery diameter. COPD phenotypes were divided as follows: A-type: absent or minor emphysema, LAA ≤ stage 1, regardless of whether the bronchial wall was thickening; E-type: the presence of LAA ≥ stage II emphysema, regardless of whether the bronchial wall was thickening; and M-type: having LAA ≥ stage II emphysema and bronchial wall thickening ≥ grade 1.

### 2.3. DNA Isolation and TNF-*α* Gene Polymorphism Typing

The TNF-*α* gene polymorphisms located on positions -308 G/A and+489 G/A, which are related to the transcriptional start site (TSS) of the TNF-*α* gene, were detected in genomic DNA derived from peripheral blood leukocytes. Briefly, 3 mL of blood was drawn from each subject after fasting and stored at -80°C after separation. Gene amplification was conducted using PCR-ABI 2700. Whole blood genomic DNA extraction was performed by Shanghai Yixiang Biotechnology Ltd. DNA was detected after cell lysis, precipitation, protease digestion, DNA adsorption, elution, and agarose gel electrophoresis. The primers for TNF-*α* -308 were designed as follows: TNF-*α*-forward (-308): 5′-AGGCCTCAGGACTCAACACA-3′; TNF-*α*-reverse (-308): 5′-GTTGCTTCTCTCCCTCTT-3′. The primers for TNF-*α* +489 were designed as follows: TNF-*α*-forward (+489): 5′-GTGTATGGAGTGAATGAATGAA-3′; TNF-*α*-reverse (+489): 5′-CCTGAGTGTCTTCTGTGT-3′. The desired gene product was isolated by agarose gel electrophoresis. PCR products were purified and sequenced by Suzhou Genewiz Biotechnology Company. If the result was bimodal at a given position, the position was interpreted as heterozygous; a single peak was interpreted as homozygous.

### 2.4. The Expression Level of TNF-*α* Based on Public Online Databases and Blood Samples

To gather the expression data of TNF-*α*, we downloaded the microarray expression profiles, including GSE71220, GSE42057, and GSE54837, from the Gene Expression Omnibus (GEO) database (https://www.ncbi.nlm.nih.gov/geo/query/acc.cgi).

The blood samples from the AECOPD group and control group were obtained in the morning on or before breakfast from the median cubital vein, then immediately centrifuged at 3,000 rpm for 10 minutes. The serum was extracted for TNF-*α* concentration assessment using TNF-*α* (human) EIA Kit (589201-480, Cayman).

### 2.5. Peripheral Blood Mononuclear Cell (PBMC) Isolation, Culture, and Differentiation

Whole blood (20-25 mL) collected in tubes containing ethylenediaminetetraacetic acid (EDTA) as anticoagulant was processed between 9 : 00 am and 12 : 00 noon, within 3 h of collection. PBMCs were isolated by Ficoll-Paque (Histopaque 1077; Sigma-Aldrich, Gillingham, UK) density gradient centrifugation (at 400 g for 30 min at RT with the break turned off) and washed twice with PBS (at 300 g for 10 min at 4°C). PBMCs were resuspended at 2.5 × 10^6^ cells/mL in RPMI 1640 medium containing 10% FCS and 1% penicillin-streptomycin. Then, the PBMCs were cultured in 24-well plates (500 *μ*l/well) and stimulated for 4 h with phorbol 12-myristate 13-acetate (PMA; 100 ng/ml; Sigma-Aldrich) and ionomycin (1 *μ*g/ml; Sigma-Aldrich) in the presence of 1× monensin (BioLegend, UK) to obtain macrophage. The culture supernatants of macrophage were collected, centrifuged for 5 min at 300 g at 4°C, and aliquoted and stored at -80°C until analysis.

### 2.6. Statistical Analysis

Data were analyzed and graphed using SPSS 19.0 statistical software (SPSS, Chicago, IL, USA). Data significance was measured using a *t*-test and *χ*^2^ test. The Hardy-Weinberg equilibrium was used to calculate the genotypic equilibrium, and the Fisher exact test was used to compare differences between genotype and allele frequency; *P* < 0.05 was considered as statistically significant.

## 3. Results

### 3.1. Characteristics of the AECOPD Populations

The AECOPD cohort consisted of 198 Shanghai Han patients (79.76 ± 8.56 years old). Around 81% of the AECOPD patients were male. A total of 121 (61%) patients were smokers or exsmokers, and 77 patients (39%) stated that they had never smoked.

The healthy population consisted of 195 Shanghai Han subjects derived from an anonymous panel of blood donors (81.26 ± 9.29 years old), including 157 males (81%) and 38 females (19%). The statistical analysis revealed no significant difference between the AECOPD cohort and the control subjects in terms of age and gender (Table 1).

### 3.2. TNF-*α* Gene Polymorphism and Population Susceptibility to AECOPD

To assess the differences of AECOPD susceptibility between the patients and control group, the genotype frequencies of TNF-*α* -308G/A and the TNF-*α* +489G/A gene polymorphisms in the two cohorts were analyzed, respectively (Table 2). As a result, in both healthy subjects and patients with AECOPD, the alleles at the individual loci of the TNF-*α* gene were in Hardy-Weinberg equilibrium, with nonsignificant *χ*^2^ values.

For TNF-*α* -308G/A gene polymorphism, the frequencies of the 3 genotypes (GG, GA, and AA) in the AECOPD group were 87.37%, 10.61%, and 2.02%, respectively, while the occurrence rates of the 3 genotypes in the control group were 95.38%, 4.62%, and 0%, respectively. Besides, the frequencies of G and A in the AECOPD group were 92.68% and 7.32%, while the frequencies in the control group were 97.69% and 2.31%, respectively. The results showed a significant difference in genotype frequency of TNF-*α* between the AECOPD group and control group (*P* = 0.001), ascribing to the enhanced frequencies of GA and AA in the patient group.

For TNF-*α* +489G/A gene polymorphism, the frequencies of the 3 genotypes (GG, GA, and AA) in the AECOPD group were 70.71%, 23.23%, and 6.06%, respectively, while the frequencies of the 3 genotypes in the control group were 75.90%, 20.00%, and 4.10%, respectively. The statistical analysis revealed that there was no significant difference in the occurrence distribution of the 3 TNF-*α* genotypes between the two groups (*P* = 0.455, R by C table Chi square test). Besides, the frequencies of G and A in the AECOPD group were 82.32% and 17.68%, while the frequencies of G and A in the control group were 85.90% and 14.10%, respectively. There was also no significant difference discovered in the occurrence distribution of the 3 TNF-*α* genotypes between the two groups (*P* = 0.455, R by C table Chi square test).

### 3.3. Correlation Analyses of TNF-*α* Gene Genotypes and AECOPD Phenotypes

**Figure 1:**
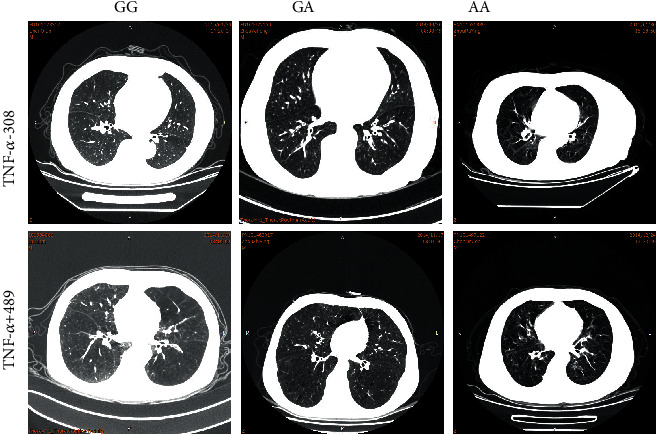
Differences among AECOPD patients with different genotypes on inspiratory-expiratory high-resolution computerized tomography (HRCT) imaging.

To investigate the correlation of TNF-*α*-308 or+489 genotypes and AECOPD phenotypes, the adjacent arterial diameter and bronchial wall thickness of AECOPD patients were measured (Figure 1 and Table 3). As a result, we found that no matter for TNF-*α* -308 or TNF-*α* +489, the patients with non-GG genotypes showed lower adjacent arterial diameter and higher bronchial wall thickness (*P* < 0.05). Two key indexes of the character of AECOPD, the ratio of bronchial wall thickness to adjacent artery diameter (bronchial artery ratio) and bronchial wall grading, were calculated. The results showed that the patients with non-GG genotypes (TNF-*α*-308 and TNF-*α* +489) had higher bronchial artery ratio and bronchial wall grading, suggesting more serious AECOPD phenotypes (*P* < 0.05).

Furthermore, for TNF-*α* -308, patients carrying non-GG genotypes showed significantly more frequent exacerbations (*P* = 0.001) and dyspnea (*P* = 0.007) than patients carrying GG genotype. Unlike TNF-*α* -308, there was no significant difference between the TNF-*α* +489AA/GA group and GG group in frequency of acute exacerbations (*P* = 0.324), while the patients with AA/GA genotype presented a higher ratio of cough to dyspnea than patients with GG genotype (*P* = 0.001). The detailed data were presented in Table 4.

### 3.4. TNF-*α* Gene Genotypes Affect the Expression Level of TNF-*α* in Macrophage

**Figure 2:**
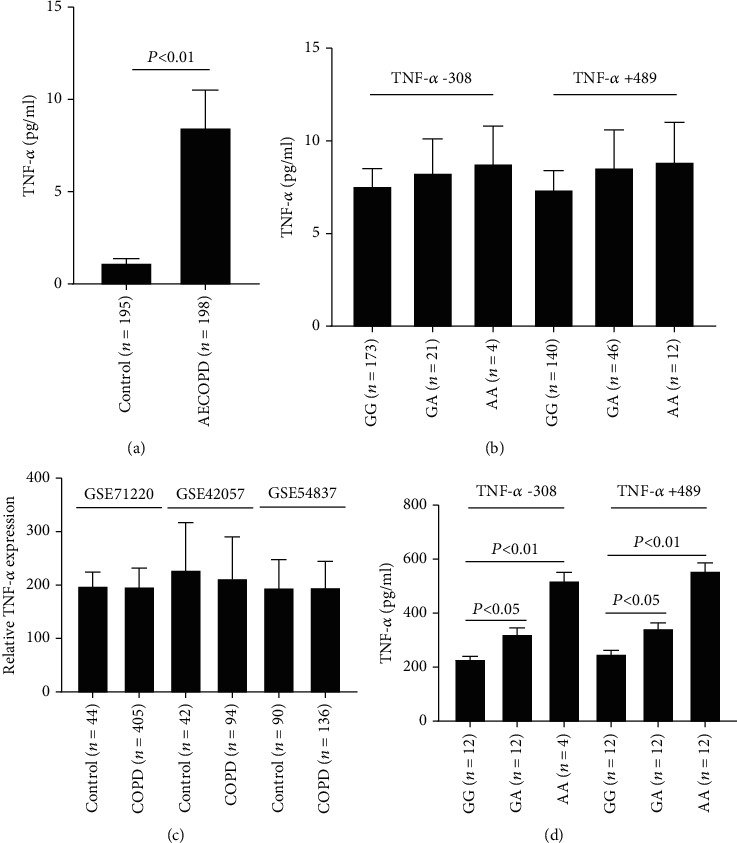
TNF-*α* gene genotypes affect the expression level of TNF-*α* in macrophage. (a) TNF-*α* concentration is increased in serum from AECOPD patients. (b) TNF-*α* concentration in serum from AECOPD patients with different TNF-*α* gene genotypes. (c) The expression of TNF-*α* in the data from public online databases. (d) TNF-*α* secreted by macrophages with different TNF-*α* gene genotypes.

To investigate whether the TNF-*α* gene genotypes affect its expression level, we firstly detected the level of TNF-*α* in blood samples of the AECOPD group and control group. As a result, TNF-*α* was overexpressed in the AECOPD group (Figure 2(a)), and the expression level was closely correlated with the TNF-*α* gene genotypes (Figure 2(b)). However, there were no significant differences in TNF-*α* expression values in three different microarray expression profiles, including GSE71220, GSE42057, and GSE54837 (Figure 2(c)). These results indicated that TNF-*α* in blood was derived from other cell types rather than lung cells. Considering that macrophage exhibited a more extensive distribution along airways, lung parenchyma, and bronchoalveolar lavage (BAL) fluid in COPD patients [30], we hypostasized that the elevation of the expression value of TNF-*α* in blood was mainly due to the extensive distribution of the macrophage population in the lung, and TNF-*α* gene genotypes could affect the expression of TNF-*α* in the lung macrophages. To verify our hypothesis, the PBMCs were isolated from blood samples of AECOPD patients carrying different TNF-*α* gene genotypes. Then, the cells were cultured and differentiated into macrophage-like cells using PMA. The TNF-*α* concentration in the culture medium was detected. The result showed that the TNF-*α* concentration was the highest in the TNF-*α* -308AA group (Figure 2(d)). A similar result was obtained for the TNF-*α* +489AA group (Figure 2(d)). These results demonstrated that TNF-*α* gene genotypes affect the expression of TNF-*α* in macrophages.

## 4. Discussion

The clinical manifestations of COPD are heterogeneous. In recent years, identifying differences between COPD phenotypes, which could facilitate the exploration of different diagnosis and treatment methods, is becoming a popular topic. The conventional drug treatment for COPD yields suboptimal responses in many patients. However, they often suffer from increasing risks of exacerbations, rapid progression, and poor prognosis. Researchers have identified and classified COPD into different categories and form different archetypes [31], which could predict the prognosis of the disease and guide the treatment for different patients.

It is now widely recognized that COPD is a chronic inflammatory disease, in which multiple inflammatory cells, inflammatory mediators, cytokines in the lung parenchyma, and pulmonary vasculature that ultimately extends to multiple systems in the body are involved. In recent years, TNF-*α* has drawn attention on account of its role in the pathogenesis of COPD. Previous studies showed that the TNF-*α* level is increased in the muscle biopsy, serum, bronchoalveolar lavage fluid (BALF), bronchial biopsy, and sputum samples of patients with COPD and associated with an elevated risk of acute exacerbation. These findings suggest that TNF-*α* is potentially involved with a local and systemic inflammatory response in the COPD process and may play an important role in determining the severity of the disease. Our previous study detecting TNF-*α* in the exhaled breath condensate of patients with AECOPD yielded similar results. Thus, TNF-*α* is one of the most active inflammatory cytokines in AECOPD. The genetic factor determines the changes in protein structures, which corresponds with pathophysiological changes. Therefore, exploring the relationship of heterogeneity of COPD phenotypes and genotype is necessary and is becoming the future trend for identifying the individualized prevention and treatment programs. The gene coding for TNF-*α* locates in the histocompatibility complex III region on chromosome 6.

Our study supported the previous reports about the role of TNF-*α* -308 gene polymorphism in AECOPD susceptibility. Meanwhile, our current study also showed that the TNF-*α* +489 GA and AA genotypes did not correlate with AECOPD susceptibility in Chinese patients of the Shanghai Han ethnicity, under the results of Hegab et al. [32], while contradictory to the opinion suggested by Kucukaycan et al. [33]. The main clinical manifestations, including dyspnea, frequent exacerbations, and significantly thickened bronchial wall, were shown by CT. Both our study and the study conducted by Kucukaycan et al. [33] yielded similar results. Among the North Caucasian population, the TNF-*α* +489G/A gene polymorphism was correlated to a higher risk of developing breathing problems. This was especially true for patients with COPD without radiographic manifestations of emphysema. Matheson et al. [34] also described that, in the Australian COPD population, patients carrying the TNF-*α* +489AA allele had a higher risk of developing breathing problems, which was associated with small airway diseases. Han et al. [35] found a 10% increase in bronchial wall thickness and a 1.42-fold increase in the number of exacerbations, suggesting that the thickening of the bronchial wall can affect clinical symptoms. Hurst et al. [27] described that acute exacerbation itself was the best predictor for disease exacerbations. Overall, COPD patients with more severe disease had a higher risk of acute exacerbation, lower quality of life, and worse disease prognosis. These findings suggested that the race, sample size, and polygenic variance of COPD could all contribute to the disease prognosis and should be validated in future clinical studies. However, the sample size of this study is small, and animal model tests are needed to further verify the results of the study. In future research, we will further explore the regulation of TNF-*α* -308 and+489 on macrophages in AECOPD.

This study included subjects of the Shanghai Han ethnicity and excluded those with hypertension, diabetes, coronary heart disease, rheumatism, and other genetic diseases. Our hypothesis for the association of the gene polymorphisms and the clinical phenotypes is that the TNF-*α* -308 and+489 locus G > A mutation may result in a higher expression value of TNF-*α* in macrophage. As a proinflammatory factor, TNF-*α* can induce the onset of COPD. The inflammation in the airway leads to a quick infiltration of neutrophils and promotes neutrophil cell adhesion, elastic peripheral cell dissociation, and proteolytic enzyme activity, which are conducive to the formation of emphysema. TNF-*α* could also induce the secretion of endothelin-1 by airway smooth muscle cells, resulting in the contraction of airway smooth muscle and airway cell proliferation, causing airway remodeling. TNF-*α* also promotes the production of IL-6 and IL-8 by bronchial epithelial cells and alveolar macrophages, thereby increasing the damage to lung tissues. In sum, TNF-*α* -308 and+489 gene polymorphisms are associated with disease severity and poor prognosis of patients with AECOPD in the Chinese Shanghai Han ethnicity. The results laid a theoretical foundation for AECOPD prevention, assessment, and personalized treatment development, thus improving the prognosis of the disease.

## Figures and Tables

**Table 1:** Clinical baseline data comparison for the AECOPD and the control group.

Groups	Gender		Age (years)
	M	F	
AECOPD	159	39	79.76 ± 8.56
Control	157	38	81.26 ± 9.29
*P* value	0.958		0.06

**Table 2:** Distribution of TNF-*α*-308 and+489 genotypes in the AECOPD and control groups.

Group	No. of samples	Genotypes			Alleles	
		GG (%)	GA (%)	AA (%)	G (%)	A (%)
*TNF-α-308*						
AECOPD	198	173 (87.37)	21 (10.61)	4 (2.02)	367 (92.68)	29 (7.32)
Control	195	186 (95.38)	9 (4.62)	0 (0)	381 (97.69)	9 (2.31)
*P* value		0.001			0.001	
*TNF-α +489*						
AECOPD	198	140 (70.71)	46 (23.23)	12 (6.06)	326 (82.32)	70 (17.68)
Control	195	148 (75.90)	39 (20.00)	8 (4.10)	329 (84.36)	61 (15.64)
*P* value		0.455			0.203	

**Table 3:** Comparison of the different indicators for the GG genotype and non-GG genotypes.

Group	No. of samples	Adjacent arterial diameter	Bronchial wall thickness	Bronchial artery ratio	Bronchial wall grading
*TNF-α-308*					
GG	173	1.04 ± 0.59	0.17 ± 0.04	0.28 ± 0.11	0.38 ± 0.55
Non-GG	25	0.65 ± 0.20	0.27 ± 0.14	0.38 ± 0.14	0.90 ± 0.75
*P* value		0.037	0.014	0.017	0.022
*TNF-α +489*					
GG	116	0.96 ± 0.56	0.26 ± 0.13	0.28 ± 0.12	0.49 ± 0.59
Non-GG	82	0.74 ± 0.39	0.21 ± 0.09	0.32 ± 0.13	0.69 ± 0.72
*P* value		0.003	0.003	0.019	0.031

**Table 4:** Comparison of the different indicators for the GG genotype and non-GG genotypes.

Group	No. of samples	Frequency of acute exacerbations		Cough or dyspnea (frequency)	
		Once/year	>twice/year	Cough	Dyspnea
*TNF-α-308*					
GG	173	153	20	105	68
Non-GG	25	11	14	8	17
*P* value		0.001		0.007	
*TNF-α +489*					
GG	116	93	23	14	102
Non-GG	82	60	22	27	55
*P* value		0.324		0.001	

## Data Availability

The data that support the findings of this study are available with approval from the author.

## Abbreviations

AECOPD: Acute exacerbation of COPD
COPD: Chronic obstructive pulmonary disease
HRCT: High-resolution computerized tomography
LAA: Low attenuation area
TNF-*α*: Tumor necrosis factor-alpha
TSS: Transcriptional start site
GEO: Gene Expression Omnibus
EDTA: Ethylenediaminetetraacetic acid.
